# *DEFB1* rs11362 Polymorphism and Risk of Chronic Periodontitis: A Meta-Analysis of Unadjusted and Adjusted Data

**DOI:** 10.3389/fgene.2019.00179

**Published:** 2019-03-12

**Authors:** Jun Shao, Miao Zhang, Lan Wu, Xiao-Wei Jia, Ying-Hui Jin, Xian-Tao Zeng

**Affiliations:** ^1^Department of Stomatology, Guangzhou Hospital of Integrated Traditional and West Medicine, Guangzhou, China; ^2^Center for Evidence-Based and Translational Medicine, Zhongnan Hospital of Wuhan University, Wuhan, China; ^3^Center for Evidence-Based and Translational Medicine, Wuhan University, Wuhan, China; ^4^Department of Evidence-Based Medicine and Clinical Epidemiology, The Second Clinical College, Wuhan University, Wuhan, China; ^5^Department of Nursing, HOPE School of Nursing, School of Health Sciences, Wuhan University, Wuhan, China; ^6^Department of Stomatology, Zhongnan Hospital of Wuhan University, Wuhan, China

**Keywords:** *DEFB1*, beta defensin 1, human β-defensins, chronic periodontitis, polymorphism, periodontal disease, meta-analysis

## Abstract

**Objective:** Chronic periodontitis (CP) is a growing problem that affects the worldwide population, having significant impacts on people's daily lives and economic development. Genetics is an important component in the determination of individual susceptibility to periodontal diseases. Numerous studies have been performed to investigate the association between beta defensin 1 (*DEFB1*) rs11362 polymorphism and risk of CP, but the results are still inconclusive. Therefore, we conducted this meta-analysis to ascertain whether this variation in *DEFB1* is associated with CP susceptibility.

**Methods:** The relevant studies were searched in PubMed and Chinese National Knowledge Infrastructure (CNKI) databases up to January 9, 2018. Two independent authors selected citations and extracted the data from eligible studies. Odds ratios (ORs) with their 95% confidence intervals (95% CIs) were used to assess the strength of the association.

**Results:** Seven case-control studies were included in this meta-analysis. Based on unadjusted data, there was no obvious association between *DEFB1* rs11362 polymorphism and CP risk in all genetic models (A vs. G: OR = 0.86, 95%CI = 0.61–1.20; AA vs. GG: OR = 0.83, 95% CI = 00.50–1.39; AG vs. GG: OR = 1.01, 95%CI = 0.73–1.39; AG+AA vs. GG: OR = 0.91, 95% CI = 00.74–1.11; and AA vs. AG+GG: OR = 0.83, 95% CI = 00.57–1.21); the results of adjusted data also showed no significant relationship. Subgroup analyses based on ethnicity, participants' smoking status, HWE in controls and severity of CP all revealed similar results to that of the overall analysis. Sensitivity analysis indicated the results were robust and no evidence of publication bias was found.

**Conclusions:** Our meta-analysis suggests that *DEFB1* rs11362 polymorphism may not have an important effect on the risk of CP. Further large-scale and well-designed studies are necessary to validate our conclusion in the future.

## Introduction

Periodontal disease mainly contains two types, namely, chronic periodontitis (CP) and aggressive periodontitis (AgP). According to the global burden of disease (GBD) in the world (Jin et al., 2016) and China (Zhang et al., 2017), periodontal disease is recognized as a major public health issue with important socio-economic impacts. In China, the standardized disability-adjusted life years (DALYs) of periodontal disease rose slightly from 24.7 in 1990 to 25.7 in 2013 based on the data from 2013 GBD study (Zhang et al., 2017). Existing evidence also indicates that periodontal disease may be related to many systematic diseases, such as cardiovascular disease (Zeng et al., 2017), carotid atherosclerosis (Zeng et al., 2016a), lung cancer (Zeng et al., 2016b), diabetes mellitus (Ziukaite et al., 2017) and gestational diabetes mellitus (Esteves Lima et al., 2016). Hence, it's of great importance to seek the risk factors and carry out effective prevention for the patients with periodontal disease. Until now, multiple environmental factors such as smoking, alcohol drinking, AIDS, Porphyromonas gingivalis are known to play significant roles in disease progression (Gemmell and Seymour, 2004; Pihlstrom et al., 2005; Rafiei et al., 2017). However, the prevalence of periodontal disease has not declined dramatically in recent decades despite the control of environmental factors.

With the rapid development of genetic epidemiology and genetic assay technologies, genetic polymorphisms are found to play a crucial part in the initiation and progression of periodontal disease (Zeng et al., 2015b; Wei et al., 2016; Weng et al., 2016; Da Silva F. R. P. et al., 2017; Da Silva M. K. et al., 2017). The genetic association studies prove that CP and AgP are probably two different diseases, and many gene polymorphisms like interleukin-1 beta (IL-1β) gene may be the susceptibility genes of CP (Genco and Borgnakke, 2013). The latest GWAS have offered novel insights into possible genetic influences and regulators of CP, including several promising candidate loci and genes. However, no genome-wide statistically significant loci have been reported to date for CP or related traits (Rhodin et al., 2014). Defensins are key elements of innate immune system and widely exist in the oral environment. Nowadays, over 30 human beta-defensin genes (*DEFB*) have been identified, some of which are expressed in the gingival tissue (Krisanaprakornkit et al., 1998; Dale et al., 2001). The expression level of DEFB varies among individuals, and it has been proposed that this variation may be attributable to genetic differences in the *DEFB1* gene (Krisanaprakornkit et al., 1998; Saitoh et al., 2004). The *DEFB* are located at chromosome 8p23.1 and show considerable variations in copy number except for the *DEFB1*, which makes *DEFB1*accessible to straightforward association analysis of potential genetic susceptibility variants (Schaefer et al., 2010). Several single nucleotide polymorphisms (SNPs) in *DEFB1* gene have been detected to correlate with health risks, such as rs11362 polymorphism (Kocsis et al., 2008; Segat et al., 2010; Estrada-Aguirre et al., 2014). The rs11362 promoter polymorphism in *DEFB1* gene is a G to A polymorphic variant in the 5′ untranslated region (https://www.ncbi.nlm.nih.gov/snp/?term=rs11362), which forms an important nuclear factor kappa B (NF-kB) transcription-binding site (Wohlfahrt et al., 2006). A previous study had suggested that the greatest allele frequency differences were in the polymorphisms in the promoter region (Ozturk et al., 2010). In 2006, Wohlfahrt et al. performed a case-control study to explore the association between *DEFB1* rs11362 polymorphism and the risk of severe CP in Caucasians (Wohlfahrt et al., 2006); since then, many relevant studies have been published. However, the results were conflicting and there is much heterogeneity across the studies, in terms of covariates used, populations and phenotypes.

As we know, the same polymorphism may play a different role in different ethnic populations, and the interaction between genetic polymorphism and classical risk factors like smoking is also valuable to be explored. Moreover, the CP can be classified into mid CP, moderate CP and severe CP. Meta-analysis is an effective method to settle controversies arising from conflicting studies and to explore and quantify the reasons for different results, because of the combined number of participants it has much greater statistical power than any individual study. Hence, we undertook this meta-analysis through pooling these results of crude data and adjusted data (Leng et al., 2016) to obtain a more precise conclusion.

## Materials and Methods

This meta-analysis was reported according to the Preferred Reporting Items for Systematic Reviews and Meta-Analyses (PRISMA) statement (Moher et al., 2009), and ethical approval was not necessary.

### Eligibility Criteria

Cohort studies or case-control studies evaluating the risk of CP in relation to *DEFB1* rs11362 polymorphism were considered eligible if they met all of the following criteria: (1) study patients were diagnosed with CP, and control group consisted of either healthy individuals or those without periodontitis; (2) CP was identified as mid CP, moderate CP, severe CP, or two or all of these three types; (3) studies reported the frequencies of genotype distribution and/or crude odds ratios (ORs) and their 95% confidence intervals (95%CIs), adjusted ORs and their 95% CIs, or the data for calculate them; (4) if two or more studies covered the same population, we included the one containing the most comprehensive information; (5) the published language was English or Chinese, and full-texts were obtainable.

### Search Strategy

We searched PubMed and Chinese National Knowledge Infrastructure (CNKI) up to January 9, 2018 using the following search terms: “DEFB1 protein, human,” “DEFB1,” “beta-defensin-1,” “hBD-1 protein,” “hBD-1,” “beta defensin 1,” “beta-defensin 1,” “beta defensin-1”, “β-defensin 1,” “ genetic,” “polymorphism,” “polymorphisms,” “variants,” “SNP,” “mutation,” “genetic variants,” “periodontal disease,” and “periodontitis.” We also screened reference lists of all relevant reviews, studies, and published meta-analyses for additional eligible studies.

### Data Extraction

The following data were extracted from all eligible studies by two authors independently and disagreements were resolved by discussion: last name of the first author, year of publication, country and ethnicity, genotyping method, source of control, numbers, and genotyping distributions of cases and controls or crude OR and its 95%CI, adjusted OR and its 95%CI, adjusted variables, and *p*-value for Hardy-Weinberg Equilibrium (HWE) in controls (Salanti et al., 2005). If the pooled data were not directly reported, we calculated them according to the methods provided in the Cochrane Handbook for Systematic Reviews of Interventions (http://training.cochrane.org/handbook).

### Assessment of Study Quality

Quality assessment was performed according to the Newcastle-Ottawa Scale, which is a validated scale for non-randomized studies in meta-analyses (Stang, 2010). A ‘star system’ (with a maximum of nine stars) has been developed in which a study is judged on three broad perspectives: the selection of the study groups; the comparability of the groups; and the ascertainment of the exposure for case-control studies.

### Statistical Analysis

Firstly, the heterogeneity was assessed using the Cochrane *Q* and *I*^2^ statistic (Huedo-Medina et al., 2006). If both *p*> 0.1 and *I*^2^≤ 50% at the same time, we used the fixed effect model; otherwise, the random effect model was applied. For crude data, OR and its 95%CI were used to quantify the strength of association in the allele comparison (A vs. G), homozygote comparison (AA vs. GG), heterozygote comparison (AG vs. GG), dominant model (AG+AA vs. GG), and recessive model (AA vs. AG+GG). For adjusted data, we directly combined the relevant ORs and their 95%CIs according to the reported genetic models. Since allele frequencies were different in various ethnicities (Ozturk et al., 2010), ethnicity was taken into consideration during our analysis so as to avoid population stratification effects. Subgroup analyses were performed based on ethnicity, smoking status, degree of CP, and HWE status in controls. Sensitivity analysis was conducted through deleting each included study in turn. Publication bias was assessed by both funnel plots and Egger's test. All statistical analyses were completed using Review Manager (RevMan) software and Comprehensive Meta-Analysis v2.2 software.

## Results

### Characteristics of Included Studies

**Figure 1:**
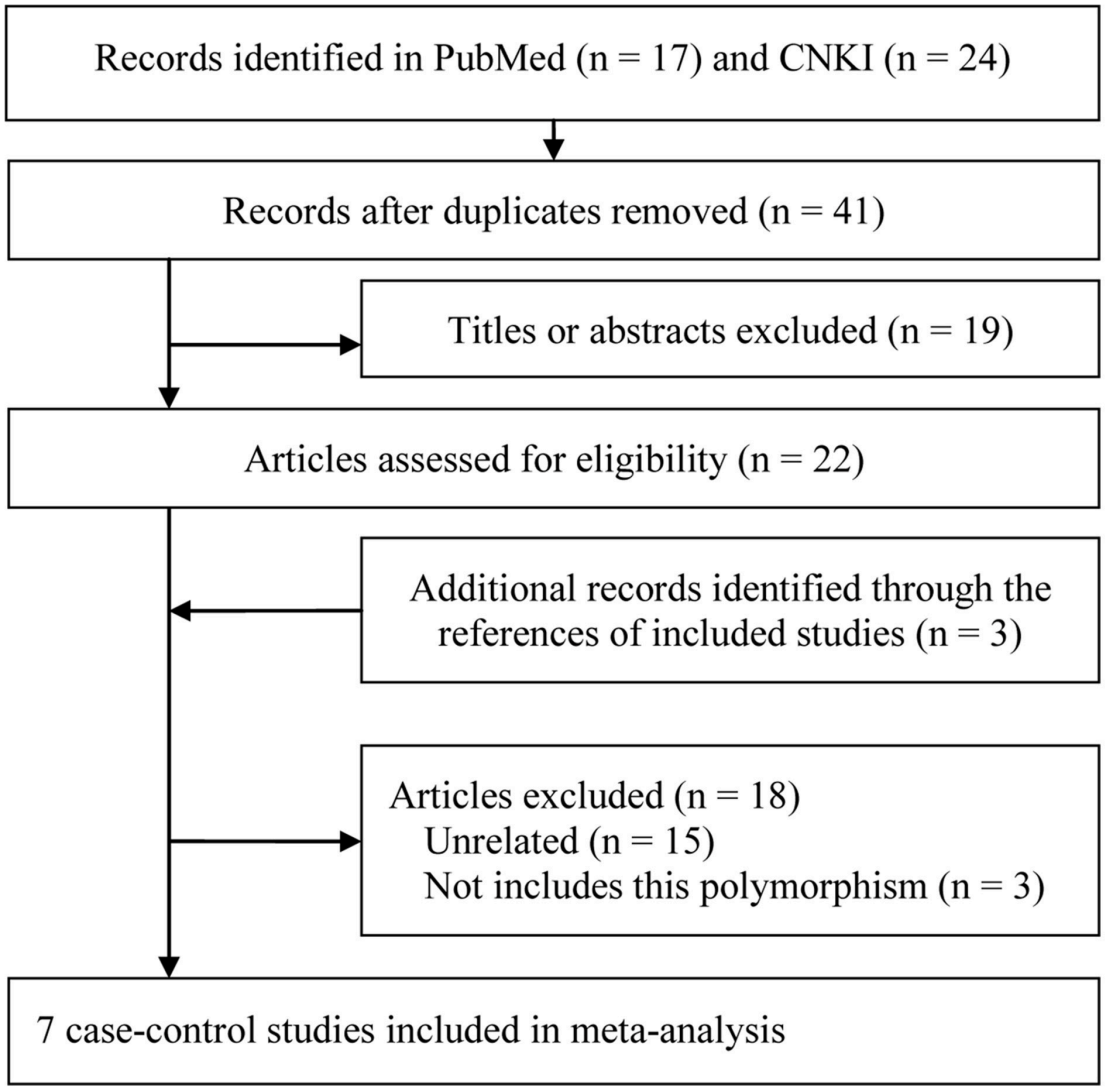
Study selection flow diagram.

A total of 41 citations were determined and of these seven case-control studies met the inclusion criteria (Wohlfahrt et al., 2006; Ozturk et al., 2010; Schaefer et al., 2010; Loo et al., 2012; Ikuta et al., 2015; Ma, 2016; Zupin et al., 2017). Figure 1 presented the process of study selection. Of them, there were five studies involving both smokers and non-smokers (Wohlfahrt et al., 2006; Ozturk et al., 2010; Schaefer et al., 2010; Ma, 2016; Zupin et al., 2017), one only including non-smokers (Loo et al., 2012) and one not report the smoking status (Ikuta et al., 2015); one study did not conform to HWE (Loo et al., 2012). Four studies provided both numbers and genotyping distributions of cases and controls as well as adjusted ORs and their 95%CIs (Wohlfahrt et al., 2006; Ozturk et al., 2010; Schaefer et al., 2010; Zupin et al., 2017). There were two studies on moderate CP (Ikuta et al., 2015; Ma, 2016) and four on severe CP (Wohlfahrt et al., 2006; Ikuta et al., 2015; Ma, 2016; Zupin et al., 2017). Detailed information of characteristics of included studies is available in Tables 1, 2. In general, the included studies were considered to be of medium to high quality, according to the Newcastle Ottawa Scale. All studies had a partial score in the selection and exposure outcomes, and four studies gained the maximum score in the comparability outcome (Table 1).

**Table 1:** Characteristics, unadjusted data, and quality assessment results of included studies.

**References**	**Country (ethnicity)**	**Type**	**Case/control**				**Source of control**	**Genotype method**	**HWE**	**Smoking status**	**Newcastle-Ottawa Scale**		
			**GG**	**GA**	**AA**	**N**					Selection (max 4 ^*^)	Comparability (max 2^*^)	Exposure (max 3^*^)
(Wohlfahrt et al., 2006)	USA (Caucasian)	SCP	47/39	62/31	28/12	137/82	Healthy	PCR	Yes	Mixed	^***^	^**^	^**^
(Ozturk et al., 2010)	USA (68% Caucasian)^#^	CP	32/76	37/91	15/36	84/203	Healthy	TaqMan	Yes	Mixed	^**^	^**^	^**^
(Schaefer et al., 2010)	Germany and Netherlands (Caucasian)	CP	187/57	304/80	115/33	606/170	Healthy	TaqMan	Yes	Mixed	^***^	^**^	^**^
(Loo et al., 2012)	China (Asian)	CP	30/38	5/12	9/58	44/108	Healthy	PCR	No	No	^***^	^*^	^**^
(Ikuta et al., 2015)	Japan (Asian)	CP	9/6	24/11	8/5	41/22	Healthy	PCR	Yes	NR	^***^	^*^	^**^
		MCP	3/6	8/11	2/5	13/22							
		SCP	6/6	16/11	6/5	28/22							
(Ma, 2016)	China (Asian)	CP	20/26	48/48	22/22	90/96	Healthy	PCR	Yes	Mixed	^***^	^*^	^**^
		MCP	11/26	23/48	12/22	46/96							
		SCP	9/26	25/48	10/22	44/96							
(Zupin et al., 2017)	Italy (Caucasian)	CP	169/41	194/80	76/34	439/155	Healthy	Illumina	Yes	Mixed	^***^	^**^	^**^
		SCP	77/41	65/80	26/34	168/155							

# *68% Caucasians, 27% African Americans, and the remaining composed of other groups; CP, Chronic periodontitis; SCP, Severe chronic periodontitis; MCP, Moderate chronic periodontitis; HWE, Hardy-Weinberg Equilibrium; Mixed, Both smokers and non-smokers; NR, Not reported*;

^*^, it stands for one star in the “star system” (with a maximum of nine stars) of the Newcastle-Ottawa Scale.

**Table 2:** Adjustment and adjusted data of included studies.

**References**	**Ethnicity**	**Type**	**Genetic models**	**OR(95%CI)**	**Adjustment**
(Wohlfahrt et al., 2006)	Caucasian	SCP	AG vs. GG	1.44 (0.70–2.93)	Gender and smoking
			AA vs. GG	1.95 (0.76–4.99)	
			(GA+AA) vs. GG	0.64 (0.33–1.23)	
			AA vs. (GA+GG)	0.62 (0.26–1.48)	
(Ozturk et al., 2010)	68% Caucasian^#^	CP	AG vs. GG	1.27 (0.7–2.3)	Age, gender, ethnicity, and smoking status
(Schaefer et al., 2010)	Caucasian	CP	AG vs. GG	1.1 (0.8–1.7)	Smoking, diabetes, and gender
			AA vs. GG	1.0 (0.6–1.7)	
			(GA+AA) vs. GG	1.1 (0.8–1.6)	
			AA vs. (GA+GG)	0.9 (0.6–1.5)	
(Zupin et al., 2017)	Caucasian	SCP	A vs. G	0.64 (0.41–0.99)	Age, gender, and smoking status

# *68% Caucasians, 27% African Americans, and the remaining composed of other groups; CP, Chronic periodontitis; SCP, Severe chronic periodontitis; OR, Odds ratio; CI, Confidence interval*.

### Overall, Heterogeneity and Sensitivity Analyses

**Figure 2:**
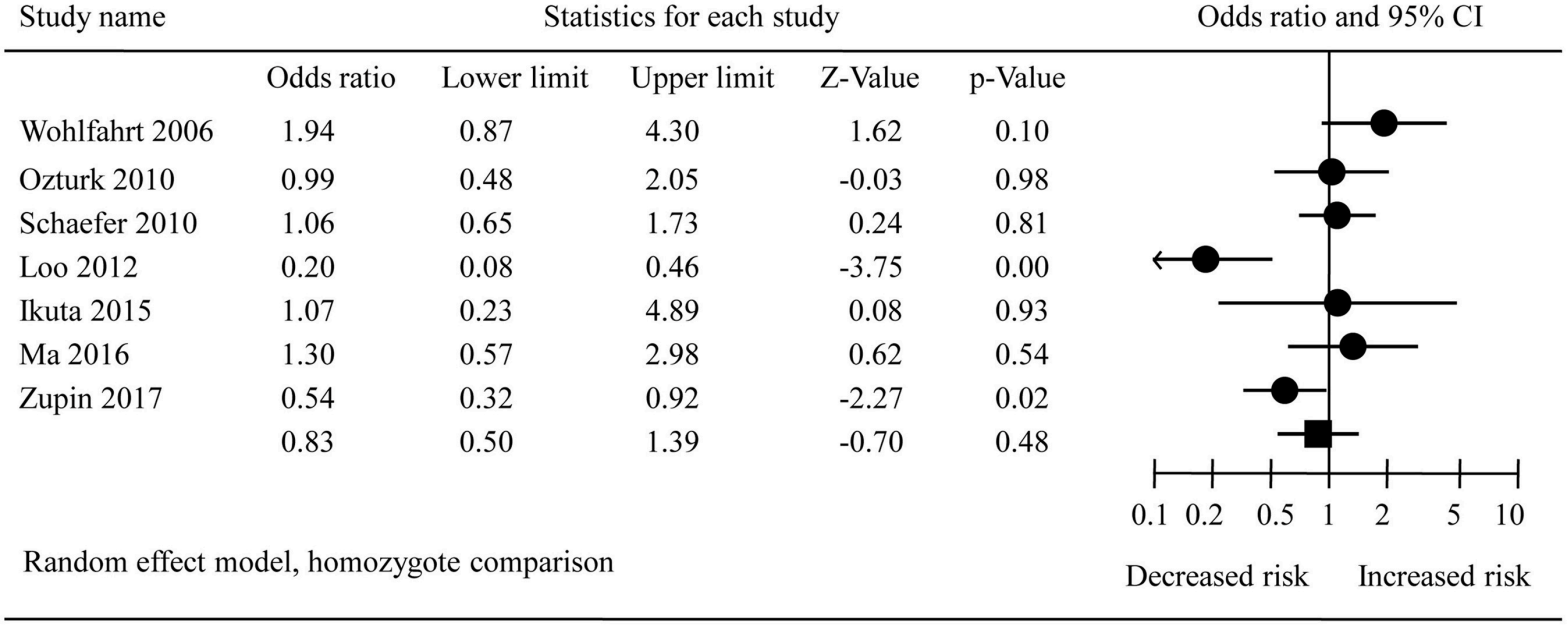
Forest plot of overall analysis in homozygote comparison. CI, Confidence interval.

**Figure 3:**
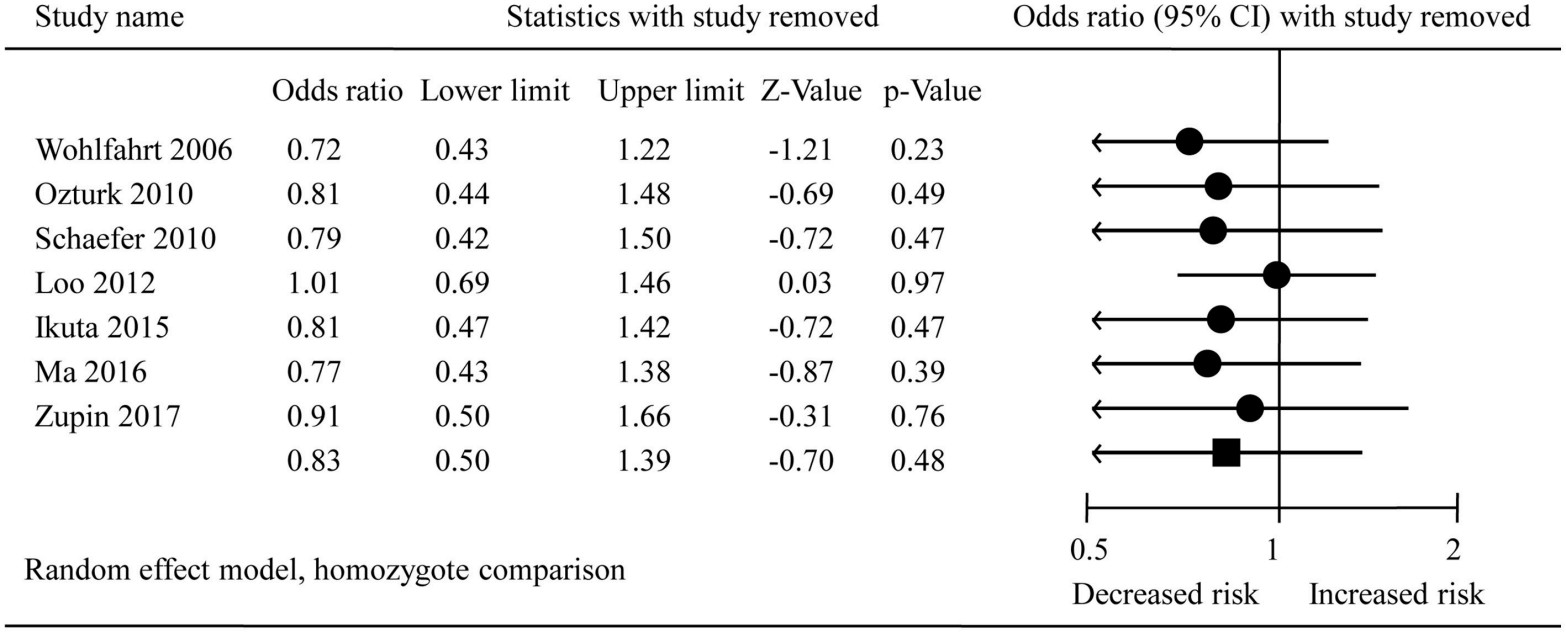
Sensitivity analysis plot of overall analysis in homozygote comparison. CI, Confidence interval.

**Figure 4:**
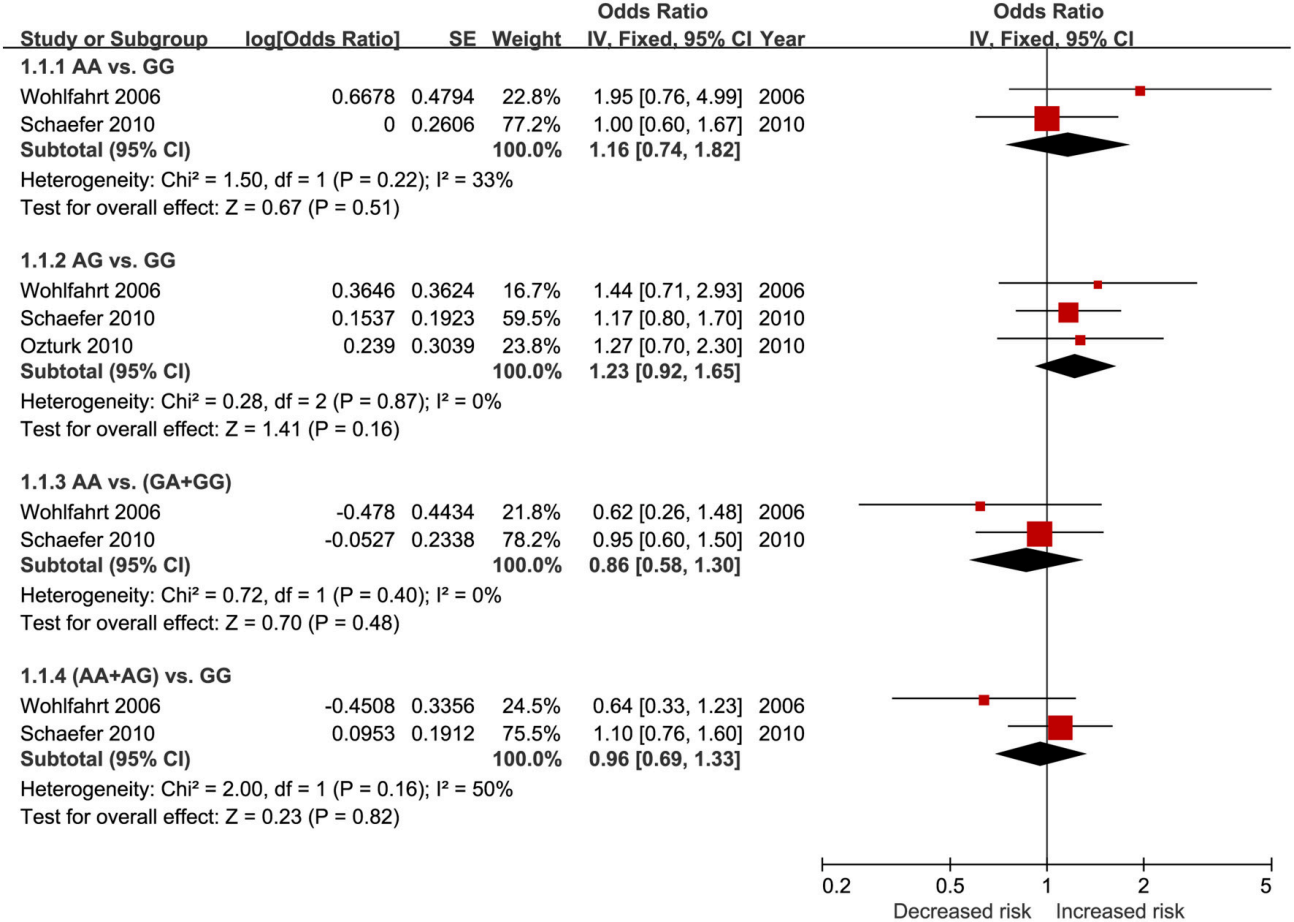
Forest plot of adjusted data in all five genetic models. CI, Confidence interval.

The pooled results from crude data indicated there was no remarkable association between *DEFB1* rs11362 polymorphism and CP risk in all genetic models [A vs. G: OR = 0.86, 95% CI = 00.61–1.20; AA vs. GG: OR = 0.83, 95% CI = 00.50–1.39 (Figure 2); AG vs. GG: OR = 1.01, 95% CI = 00.73–1.39; AG+AA vs. GG: OR = 0.91, 95% CI = 00.74–1.11; AA vs. AG+GG: OR = 0.83, 95% CI = 00.57–1.21]. The heterogeneity of all the five genetic models were significant, so the results were pooled using random effects model. All the results are presented in Table 3. Sensitivity analysis indicated that the findings remained unchanged (Figure 3). The results of adjusted data also showed no association between DEFB1 rs11362 polymorphism and CP risk, except that a boundary relationship was observed in A vs. G genetic model (Figure 4).

**Table 3:** Overall and subgroups meta-analysis of unadjusted data.

**Overall and subgroup analysis**	**No. of studies**	**OR(95%CI)**	***I^**2**^*(%)/ *p***
**A vs. G**			
Total	7	0.86 (0.61–1.20)	81.7/ <0.001
Asian	3	0.66 (0.24–1.84)	90.43/ <0.001
Caucasian	3	0.99 (0.69–1.42)	77.41/ 0.012
68% Caucasians^#^	1	0.99 (0.69–1.43)	NA
HWE (No)	1	0.24 (0.14–0.42)	NA
HWE (Yes)	6	1.00 (0.82–1.23)	47.83/ 0.088
Smoking (Mixed)	5	1.00 (0.80–1.26)	58.11/ 0.049
Non-smoker	1	0.24 (0.14–0.42)	NA
Smoking (NR)	1	1.04 (0.50–2.17)	NA
Moderate CP	2	1.09 (0.70–1.70)	0.00/ 0.733
Severe CP	4	0.98 (0.61–1.59)	76.51/ 0.005
**AA vs. GG**			
Total	7	0.83 (0.50–1.39)	70.41/ 0.002
Asian	3	0.62 (0.16–2.39)	80.90/ 0.005
Caucasian	3	0.99 (0.51–1.91)	70.33/0.024
68% Caucasians^#^	1	0.99 (0.48–2.05)	NA
HWE (No)	1	0.20 (0.08–0.46)	NA
HWE (Yes)	6	0.96 (0.73–1.27)	38.61/ 0.148
Smoking (Mixed)	5	1.01 (0.67–1.53)	50.77/ 0.087
Non-smoker	1	0.20 (0.08–0.46)	NA
Smoking (NR)	1	1.07 (0.23–4.89)	NA
Moderate CP	2	1.18 (0.48–2.92)	0.00/ 0.693
Severe CP	4	1.00 (0.42–2.39)	70.12/ 0.018
**AG vs. GG**			
Total	7	1.01 (0.73–1.39)	46.31/ 0.083
Asian	3	1.09 (0.63–1.87)	0.00/ 0.373
Caucasian	3	1.02 (0.57–1.80)	77.91/ 0.011
68% Caucasians^#^	1	0.97 (0.55–1.70)	NA
HWE (No)	1	0.53 (0.17–1.66)	NA
HWE (Yes)	6	1.05 (0.75–1.47)	50.00/ 0.075
Smoking (Mixed)	5	1.04 (0.72–1.48)	58.59/ 0.047
Non-smoker	1	0.53 (0.17–1.66)	NA
Smoking (NR)	1	1.45 (0.41–5.11)	NA
Moderate CP	2	1.19 (0.56–2.57)	0.00/0.793
Severe CP	4	1.06 (0.47–2.39)	78.29/0.003
**AA vs. (AG+GG)**			
Total	7	0.83 (0.57–1.21)	56.93/ 0.030
Asian	3	0.58 (0.20–1.70)	77.33/ 0.012
Caucasian	3	0.93 (0.70–1.24)	21.70/ 0.279
68% Caucasians^#^	1	1.01 (0.52–1.96)	NA
HWE (No)	1	0.22 (0.10–0.51)	NA
HWE (Yes)	6	0.96 (0.75–1.22)	0.00/ 0.731
Smoking (Mixed)	5	0.96 (0.75–1.23)	0.00/ 0.601
Non-smoker	1	0.22 (0.10–0.51)	NA
Smoking (NR)	1	0.82 (0.23–1.91)	NA
Moderate CP	2	1.06 (0.51–2.23)	0.00/0.519
Severe CP	4	0.91 (0.62–1.33)	3.98/0.373
**(AA+AG) vs. GG**			
Total	7	0.91 (0.74–1.11)	75.25/ <0.001
Asian	3	0.74 (0.23–2.36)	82.61/ 0.003
Caucasian	3	1.02 (0.56–1.85)	82.24/ 0.004
68% Caucasians^#^	1	0.97 (0.58–1.64)	NA
HWE (No)	1	0.25 (0.12–0.53)	NA
HWE (Yes)	6	1.06 (0.75–1.49)	58.97/ 0.032
Smoking (Mixed)	5	1.04 (0.71–1.52)	66.55/ 0.018
Non-smoker	1	0.25 (0.12–0.53)	NA
Smoking (NR)	1	1.33 (0.40–4.40)	NA
Moderate CP	2	1.20 (0.58–2.47)	0.00/0.951
Severe CP	4	1.05 (0.45–2.44)	82.00/0.001

# *68% Caucasians, 27% African Americans, and the remaining composed of other groups; CP, Chronic periodontitis; OR, Odds ratio; CI, Confidence interval; HWE, Hardy-Weinberg Equilibrium; Mixed, Both smokers and non-smokers; NR, Not reported; NA, Not applicable*.

### Subgroup and Publication Bias Analyses

**Figure 5:**
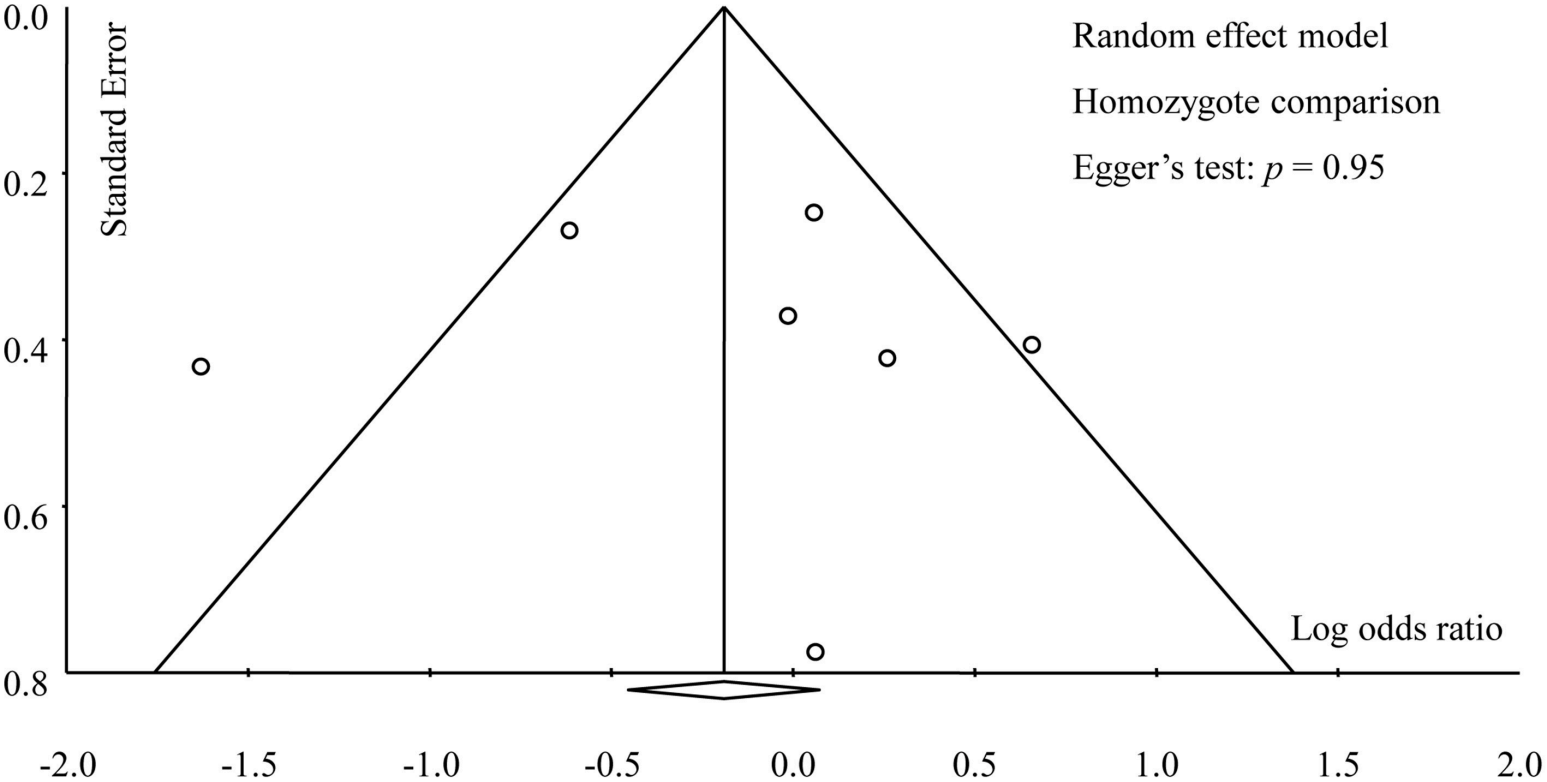
Funnel plot of overall analysis in homozygote comparison.

Table 3 provided the results of all subgroup analyses, which were similar to that of the overall analysis. Dependably, no evidence of publication bias was found in this meta-analysis in any genetic models, which was supported by Egger's test (A vs. G: *p* = 0.68; AA vs. GG: *p* = 0.95; AG vs. GG: *p* = 0.78; AA vs. AG+GG: *p* = 0.70; AA+AG vs. GG: *p* = 0.94) and symmetric funnel plots (Figure 5).

## Discussion

With an increasing prevalence of CP in the developing world (Albandar and Rams, 2002), it's urgent and significant to prevent and control the disease. Besides microbial colonization (Nibali et al., 2007) and environmental factors (such as smoking) (Barbour et al., 1997), genetic mutations are also considered as a risk factor in the pathogenesis of CP (Vijayalakshmi et al., 2010). Defensins comprise a large group of different peptides with 3 disulphide bonds; in the oral cavity, the α- and β-defensins exist in the epithelia, involving gingiva (Krisanaprakornkit et al., 1998; Dale et al., 2001), sulcular fluid (Ganz et al., 1985), saliva, and salivary glands (Zhao et al., 1996; Bonass et al., 1999). They are at high concentrations in healthy and inflamed tissues of the whole body, possibly constituting the first line of defense against pathogens in the mouth (Navarra et al., 2016). Sinceoral cavity has been recognized as a potential reservoir for respiratory pathogens (Mojon, 2002), dental plaque may play an important role in this accident, contributing to periodontal disease (Coulthwaite and Verran, 2007). Hence, periodontal disease has been identified as an inflammatory disease, especially CP. In the past decade, some investigators have paid attention to the correlation between defensins and CP. DEFB1 is a prominent molecule of the defense in family and there are several lines of evidence supporting its potential role in periodontal diseases. For instance, induction of *Aggregatibacteractinomycetemcomitans*, a periopathogenic bacterium, was found to increase DEFB1 levels in gingival epithelium (Kurland et al., 2006).

Previous work on cancer cell lines suggests that promoter methylation of *DEFB1* may not play an important role in *DEFB1* gene regulation (Sun et al., 2006). It is well-accepted that genetic studies of periodontal disease have the potential to lead to a better understanding of the etiopathogenesis of periodontal disease, although the inherited risk variants have largely remained unexplained. In 2006, Wohlfahrt et al. found no obvious association between *DEFB1* rs11362 polymorphism and risk of severe CP in Caucasians (Wohlfahrt et al., 2006); a study by Ozturk et al. in 2010 confirmed no important effect of *DEFB1* genetic markers on periodontal disease (Ozturk et al., 2010). This is the first meta-analysis comprehensively performed to investigate the relationship between *DEFB1* rs11362 polymorphism and risk of CP. Our meta-analysis of seven case-control studies also indicated no significant association between this variant and the susceptibility of CP, either based on unadjusted or adjusted data. Unlike the usual method based on unadjusted data (Zeng et al., 2015b; Wei et al., 2016; Weng et al., 2016; Da Silva F. R. P. et al., 2017; Da Silva M. K. et al., 2017), we also extracted the adjusted data and pooled them to detect the interactions between genetic polymorphism and environmental risk factors. Considering that smoking as a risk factor might influence the treatment of periodontitis (Fiorini et al., 2014; Kotsakis et al., 2015), subgroup analysis by smoking status was conducted and the results suggested that this polymorphism was not affected by smoking status (Table 3). Additionally, when data were analyzed with adjustment for gender and smoking, the association also remained insignificant (Table 2). The study by Schaefer et al. (2010) provided the adjusted data for diabetes, which had a bidirectional relationship with periodontal disease, demonstrating no statistical association likewise. Moreover, subgroup analyses based on ethnicity, HWE in controls and severity of CP revealed similar results to that of the overall analysis.

The major strength of this meta-analysis was that we considered adjusted data and severity of CP. However, as we know, meta-analysis is a secondary research, the results of which largely depend on the data from original studies (Zeng et al., 2015a), so some inherent limitations existed in our research. Firstly, our meta-analysis only included seven case-control studies and the sample size of each study was relatively small. Limited sample sizes would influence the statistical power, thereby resulting in false negative or false positive result probably. Although our meta-analysis revealed a non-significant association, we could not know whether this was the final result or not due to the small sample sizes. Secondly, limited by the ability of language and uses-permission of databases in many countries, we could not retrieve all databases. Hence, studies published in such languages as Russian, Japanese, Korean, Iranian, German, and Spanish might be missed in this meta-analysis. Although no large publication bias was inspected in this work, we would not ignore its influence. Thirdly, considering the time cumulative effect, the ratios of the effects of *DEFB1* rs11362 polymorphism on CP ranging from mild to severe were different. Thus, we performed stratified analysis based on the severity of CP. However, only data of moderate and severe CP were extracted because the data of mild CP was not reported in the included studies, which limited us to performing the dose-response analysis.

Fourthly, allele frequencies were different between Caucasians and African Americans as reported by Ozturk et al. The difference in different allele frequencies between populations would suggest a different haplotype linkage disequilibrium and therefore different haplotype structure between ancestral populations. Although ethnicity based subgroup analysis was conducted, our results could also be influenced by undetected population stratification. In the periodontal researches, there is a common issue that different studies use different diagnosis criteria. There is more than one accepted diagnosis criterion of periodontal disease, such as alveolar bone loss, oral health index, periodontal index and clinical attachment loss; moreover, the detailed information of diagnosis was not reported usually in the published studies. Consequently, our results should be interpreted with caution.

In consideration of the above-mentioned restrictions in our meta-analysis, large-scale studies with detailed information of smoking, alcohol drinking, diagnosis criteria, disease severity, ethnic background, concomitant diseases, and occupation are required to be conducted, so as to yield more precise results of the association between *DEFB1* rs11362 polymorphism and CP. Moreover, the difference between smokers and non-smokers is also needed to be detected, thereby providing evidence to ascertain the real role of *DEFB1* rs11362 polymorphism and smoking in the occurrence and development of CP.

## Author Contributions

JS and X-TZ designed this study. MZ and LW searched databases and collected full-text papers. MZ and Y-HJ extracted and analyzed data. JS, X-WJ, and LW wrote the manuscript. X-TZ reviewed the manuscript.

### Conflict of Interest Statement

The authors declare that the research was conducted in the absence of any commercial or financial relationships that could be construed as a potential conflict of interest.
